# Psycho-Oncology in Breast Cancer: Supporting Women Through Distress, Treatment, and Recovery—Three Arguments—Rapid Narrative Review

**DOI:** 10.3390/medicina61061008

**Published:** 2025-05-28

**Authors:** Mădălina Daniela Meoded, Mariana Tănase, Claudia Mehedințu, Ciprian Cirimbei

**Affiliations:** 1 “Carol Davila” University of Medicine and Pharmacy, 050474 Bucharest, Romania; madalina-daniela.oancea@drd.umfcd.ro; 2 Outpatient Psychology Department, Bucharest Institute of Oncology “Prof. Dr. Al. Trestioreanu”, 022328 Bucharest, Romania; 3 Faculty of Psychology and Educational Sciences, “Hyperion” University, 030615 Bucharest, Romania; 4 Department of Obstetrics and Gynaecology, “Carol Davila” University of Medicine and Pharmacy & “Filantropia” Clinical Hospital, 011684 Bucharest, Romania; claudia.mehedintu@umfcd.ro; 5 Department of Surgery, Bucharest Institute of Oncology “Prof. Dr. Al. Trestioreanu”, “Carol Davila” University of Medicine and Pharmacy, 022328 Bucharest, Romania

**Keywords:** psycho-oncology, breast cancer, quality of life, treatment adherence, post-traumatic growth

## Abstract

*Background and Objectives:* Breast cancer remains one of the most prevalent malignancies affecting women and one of the most emotionally distressing diagnoses. Emerging evidence suggests that chronic psychological stress may influence cancer progression via neuroendocrine and immune mechanisms. This rapid narrative review explores three key arguments for integrating psycho-oncologists into the multidisciplinary care of breast cancer patients: (1) early detection and reduction of psychological distress, (2) improvement of treatment adherence, and (3) enhancement of quality of life through personalized psychological interventions. *Materials and Methods:* The review was conducted through comprehensive searches in PubMed, Scopus, and Web of Science for peer-reviewed articles published between 2010 and 2025. Inclusion criteria comprised randomized controlled trials, systematic reviews, meta-analyses, and theoretical papers. Of 246 identified articles, 50 met the inclusion criteria. *Results:* Selected studies show that psycho-oncological interventions—including cognitive–behavioral therapy, mindfulness-based techniques, narrative therapy, and guided imagery—significantly reduce anxiety, depression, and fear of recurrence. These approaches improve adherence to endocrine therapy and chemotherapy, enhance emotional resilience, and promote overall well-being. Also, recent research concepts emphasize the role of psycho-oncologists in encouraging post-traumatic growth and helping patients redefine cancer as an opportunity for transformation. *Conclusions:* Integrating psycho-oncologists into standard breast cancer care improves psychological and clinical outcomes. By addressing emotional distress, strengthening coping mechanisms, and supporting existential resilience, psycho-oncologists contribute to a holistic, patient-centered model of oncology care. Wider implementation of psycho-oncological services is warranted as a core component of comprehensive cancer management.

## 1. Introduction

Breast cancer is one of the most common forms of cancer in women, having a significant impact not only on physical health but also on psychological well-being, with a global incidence of approximately 2,300,000 new cases, which places it in second place among cancers, and with a mortality of approximately 666,100 cases, which places it in fourth place among cancers, according to data provided by GLOBOCAN in 2022 [1]. While the incidence and mortality data provide a quantitative view of breast cancer’s burden, a deeper understanding requires exploring the underlying biological mechanisms contributing to tumor development and progression.

Although the exact mechanisms underlying cancer initiation remain incompletely understood, it is widely accepted that both genetic and cellular alterations, triggered by endogenous and exogenous factors, contribute to tumorigenesis. Emerging literature also explores the potential influence of psychological stress, especially following emotional trauma, in modulating biological systems such as the neuroendocrine and immune axes, which may play a role in cancer progression [2,3,4].

On the other hand, a cancer diagnosis brings with it a series of emotional challenges, such as anxiety, depression and existential distress, which can negatively affect the patient’s therapeutic journey. In these conditions, a holistic approach to patients becomes essential, combining oncological treatments with specialized psychological support.

Given this intricate mind–body interplay, the emerging field of psycho-oncology provides a necessary framework for addressing not only the psychological impact of cancer but also its potential reciprocal influence on disease biology. Psycho-oncology focuses on understanding and managing the psychological impact of cancer on patients, and on the psychological conditions that may generate and promote cancer. In the case of breast cancer patients, the intervention of a psycho-oncologist can bring significant benefits by detecting subtle signs of psychological distress that may contribute to disease initiation or progression, and consequently by reducing emotional distress and improving treatment adherence and increasing quality of life [5,6].

Despite growing recognition of the psychological impact of cancer, many reviews tend to generalize psycho-oncological principles across all cancer types, often overlooking the specific vulnerabilities and psychosocial trajectories unique to breast cancer patients. Breast cancer, due to its symbolic and physical implications related to femininity, body image, and identity, requires tailored psycho-oncological strategies. This narrative review addresses a timely and urgent need: to synthesize the evidence specific to breast cancer and to highlight concrete, argument-based justifications for integrating psycho-oncologists into routine care. By focusing exclusively on breast cancer, the review aims to fill a gap in the literature and offer a structured, clinically relevant framework for implementation.

This narrative review seeks to clarify and support the inclusion of psycho-oncological care as an integral component of breast cancer management. We present three interrelated arguments—focusing on psychological distress, treatment adherence, and quality of life—to build a coherent perspective on why psycho-oncology should be embedded in multidisciplinary oncology teams. Each section is structured to illustrate how specific interventions address practical clinical challenges, offering both scientific evidence and real-world applicability.

Although psycho-oncology is a growing field, we emphasize that its implementation in routine breast cancer care remains limited, particularly in Eastern European systems.

## 2. Materials and Methods

This study was designed as a rapid narrative review, aiming to synthesize the most relevant evidence regarding the psychological benefits of psycho-oncological interventions in breast cancer patients. This review exclusively included studies focused on breast cancer due to the distinct psychosocial burden associated with this diagnosis. Compared to other malignancies, breast cancer presents unique psychological dimensions—including body image disruption, identity reconstruction, and the impact on femininity and sexuality—which necessitate targeted psycho-oncological approaches. The inclusion criteria were therefore deliberately restricted to interventions addressing psychological distress in women with breast cancer.

### 2.1. Search Strategy

We conducted a literature search in three major databases, PubMed, Scopus, and Web of Science, covering the period January 2010–March 2025. The search terms used in various combinations included “breast cancer”, “psycho-oncology”, “psychological intervention”, “quality of life”, “emotional distress”, “treatment adherence”, “mindfulness”, “cognitive-behavioral therapy”, and “post-traumatic growth”. The search was limited to English-language publications involving human subjects.

Two reviewers independently screened titles and abstracts. Full texts of potentially relevant studies were then assessed for eligibility. Discrepancies were resolved by discussion. No formal risk-of-bias assessment was performed, as this was beyond the scope of a narrative review.

### 2.2. Inclusion and Exclusion Criteria

Inclusion criteria: Studies focusing on randomized controlled trials, cohort studies, meta-analyses, and systematic reviews that addressed the role of psycho-oncological support in breast cancer (e.g., anxiety, depression, quality of life, adherence to treatment).

Exclusion criteria: Non-peer-reviewed articles (e.g., conference abstracts, editorials, opinion papers), studies involving cancer populations without breast cancer-specific data, articles not available in full text, and studies focused exclusively on pediatric or male populations with breast cancer.

## 3. Results

After the initial search, a total of 246 articles were identified. Duplicates were removed, resulting in 192 unique records. Titles and abstracts were screened for relevance by two independent reviewers. Of these, 112 articles were excluded for not meeting the inclusion criteria. The remaining 80 full-text articles were assessed, and 50 articles were retained for inclusion in the final review based on their methodological rigor and thematic relevance.

**Figure 1:**
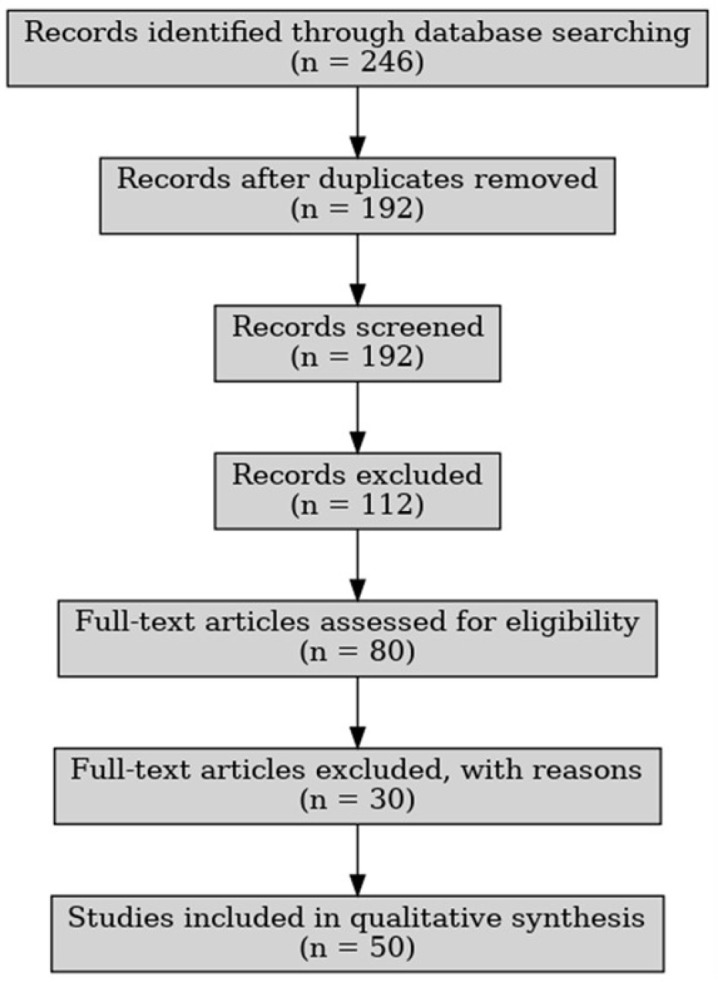
Flow diagram of article identification and inclusion process in review.

The study selection process is illustrated in this flow diagram below (Figure 1):

A qualitative thematic analysis was performed on the selected studies. Three key themes were identified and structured as the core arguments of this review: identification and reduction of emotional distress and psychological stress, improvement of treatment adherence, and enhancement of quality of life.

Where possible, evidence from meta-analyses and randomized controlled trials was highlighted to support the strength of findings.

## 4. Argument 1: Identifying Psychological Stress and Reducing Emotional Distress in Recently Diagnosed Breast Cancer Patients Through Personalized Interventions

Breast cancer represents not only a physical challenge but also a major psychological trauma. Studies show that up to 50% of women diagnosed with breast cancer develop clinical forms of anxiety and depression during the disease [7]. Untreated emotional distress can lead to difficulties in making treatment decisions, decreased quality of life, and worsening of the overall prognosis.

Chronic stress, including psychological stress, can generate physiological disorders that disturb the body’s balance, acting through various signaling pathways, particularly the neuroendocrine system and the nervous system, which can even interfere with carcinogenesis processes. Under stress conditions, there is stimulation of the hypothalamic–pituitary–adrenal (HPA) axis and the sympathetic nervous system, leading to abnormal hormone release. The classical hormonal mediators of the stress response are glucocorticoids and catecholamines. While these have beneficial, protective short-term effects—creating optimal conditions for adaptation and survival by maintaining homeostasis—in the long term, they may initiate or contribute to various diseases, including activation of signaling pathways that can increase oncogene expression [8,9,10].

In this regard, Marta Falcinelli [2] highlights the link between chronic psychological stress and cancer initiation, through complex biological mechanisms. The study emphasizes that chronic stress may induce a tumor-promoting microenvironment by suppressing T-cell- and NK-cell-mediated immunity, activating inflammatory pathways and pro-inflammatory cytokines, and increasing angiogenesis and cell migration through the release of growth factors (vascular endothelial growth factor (VEGF)). Concerning its impact on the immune system, psychological stress activates the HPA axis and the sympathetic nervous system, leading to the release of stress hormones (e.g., cortisol and epinephrine); these hormones contribute to the suppression of anti-tumor immunity, by decreasing NK cell and cytotoxic T lymphocyte activity—cells essential for the detection and elimination of tumor cells. The article proposes the hypothesis that patients exposed to chronic stress are at a higher risk of cancer development and progression, due to immunological imbalances. Another mechanism implicated in cancer progression is chronic inflammation. The article emphasizes that psychological stress can increase the levels of inflammatory cytokines such as IL-6 and TNF-α, which contribute to cancer initiation by promoting DNA mutations and increasing genomic instability. In addition to promoting mutagenesis, inflammatory mediators also modulate epigenetic mechanisms that control gene expression. Tumor necrosis factor-alpha (TNF-α), a major pro-inflammatory cytokine, stimulates the generation of reactive oxygen species (ROS) in epithelial cells, thereby contributing to genomic instability. Furthermore, cytokines such as interleukin-6 (IL-6) and interleukin-23 (IL-23) help establish a tumor-promoting microenvironment by activating key transcription factors, including nuclear factor kappa B (NF-κB) and signal transducer and activator of transcription 3. These factors drive the expression of genes linked to cell survival, proliferation, and immune escape, ultimately supporting tumor development and progression [11]. Additionally, psychological stress may trigger several molecular mechanisms that contribute to carcinogenesis. One such mechanism involves the activation of the NF-κB pathway through increased production of epinephrine and norepinephrine. These stress hormones stimulate NF-κB, a key transcription factor that regulates genes involved in inflammation, immune response, and cell survival. In mammary epithelial cells, NF-κB activation may accelerate malignant transformation. Moreover, stress-induced release of cortisol has been shown to promote angiogenesis by regulating the expression of VEGF, thereby facilitating the formation of new blood vessels essential for tumor development and progression. Chronic psychological stress is also associated with genomic instability due to elevated levels of free radicals, and can cause oxidative DNA damage, contributing to oncogenic mutations [12,13,14].

Given these complex physiological effects of stress, attention must also be paid to the psychological consequences of a cancer diagnosis itself, which often triggers anxiety, depression, and existential distress. These emotional responses can, in turn, influence disease trajectory and treatment outcomes, highlighting the importance of integrating psycho-oncological care into breast cancer management.

The importance of reducing psychological stress in the evolution of cancer is also addressed in an article published by Hyeon-Muk Oh and Chang-Gue Son [15]. The authors emphasize that chronic psychological stress has been identified as a risk factor for cancer recurrence, through various biological mechanisms cited in multiple studies, including immune dysfunction and increased inflammation—factors that can contribute to the initiation and progression of the disease. This highlights the need for early assessment and management of stress among breast cancer patients, as this may have a positive impact on prognosis by reducing systemic inflammation and promoting a more effective immune response. Psycho-oncological interventions, such as mindfulness techniques or cognitive–behavioral therapy (CBT), are essential in alleviating chronic stress, thus contributing to the reduction in recurrence risk. Considering the evidence provided by Oh and Son, reducing psychological stress should be integrated into standard treatment protocols for breast cancer patients—not only as a priority for enhancing emotional well-being but also as a potential strategy for lowering the risk of disease recurrence.

Expanding on the concept of personalized medicine in oncology, Giampaolo Perna and colleagues [16] offer a valuable perspective on personalized approaches in psycho-oncology. The authors emphasize the importance of tailoring interventions to the individual needs of breast cancer patients. They argue that “one-size-fits-all” approaches are less effective in managing emotional distress. According to their analysis, considering individual characteristics—such as personality traits, baseline mental health status, and biological factors—can significantly enhance outcomes. Perna et al. suggest that personalized psycho-oncological interventions, which are adapted to each patient’s unique profile, are considerably more effective in reducing emotional distress. Furthermore, the customization of therapeutic techniques such as cognitive–behavioral therapy or mindfulness to align with individual needs can amplify their positive impact on mental health.

The impact of the psycho-oncologist in managing emotional distress is gradually taking its rightful place in the fight against cancer. Psycho-oncologists are trained to detect the subtle signs of psychological stress that may contribute to the onset or progression of the disease. As a result, several psychological interventions have become essential in the management of patients recently diagnosed with breast cancer.

*Historical and contextual assessment* includes the identification of past traumas, emotional losses, or prolonged periods of intense stress, as well as the evaluation of acute or chronic stress factors in the patient’s life (e.g., family events, financial difficulties) [17,18]. To understand the impact of stress on patients’ health, a thorough evaluation of their personal and contextual history is essential. This includes identifying past traumatic events such as the loss of a loved one, divorce, emotional or physical abuse, or other significant experiences that have left deep emotional scars. It is also important to assess prolonged periods of intense stress, such as family conflicts, professional instability, or financial hardship.

This assessment can reveal acute stressors (e.g., recent losses) or chronic stressors (e.g., poor socio-economic conditions, discrimination) that may contribute to the patient’s psychological and physical vulnerability. Detailed conversations, conducted in an empathetic setting, help not only to identify these factors but also to build a relationship of trust between the patient and the specialist. At this stage, the specialist may use semi-structured interviews or discussion guides to explore essential aspects: How frequently and intensely does the patient experience stress? How does she respond emotionally and behaviorally to difficult situations? What coping mechanisms has she used in the past? An empathetic and confidential approach during these discussions creates a safe space for the patient, encouraging her to share sensitive information. This process not only offers a comprehensive perspective on stress sources but may also reveal key points for intervention, such as the need for family support or financial counseling [19].

*Use of Validated Instruments:* The application of validated psychological scales and questionnaires, such as the Perceived Stress Scale (PSS) and the Hospital Anxiety and Depression Scale (HADS), is essential for assessing perceived stress levels and their impact on mental health [20,21]. The PSS evaluates the extent to which a patient perceives situations in her life as stressful, providing insight into feelings of lack of control or reduced ability to cope with challenging circumstances. HADS allows for the assessment of anxiety and depression, two conditions frequently associated with chronic stress and a cancer diagnosis. This scale is particularly useful for identifying emotional states that can negatively affect both the patient’s quality of life and adherence to treatment. These instruments offer quantifiable data that can guide subsequent interventions, enabling specialists to tailor psychological support strategies and monitor the patient’s progress throughout the course of treatment.

Correlating emotional cleavage with disease onset and exploring the moment when patients began experiencing cancer symptoms alongside the stressful events that preceded the diagnosis represent critical aspects of psycho-oncological intervention [22]. For instance, the loss of a life partner, a divorce, or a traumatic event may be linked to the onset or exacerbation of symptoms. This type of exploration allows specialists to identify the mechanisms through which stress can influence a patient’s physiology, ranging from the activation of the hypothalamic–pituitary–adrenal (HPA) axis to immune or hormonal dysregulations. Understanding these connections provides a holistic perspective on illness, helping to develop integrated treatment strategies that address both the psychological and physical dimensions of health.

An ambitious study was launched in 2023 by Susana S. Almeida [23], set to span over a 10-year period, and offers valuable insight into the genetic and inflammatory factors that influence resilience and vulnerability to depression among premenopausal breast cancer patients. It supports the hypothesis that emotional stress and inflammation may contribute to the initiation or progression of the disease by altering stress-related genetic responses. Accordingly, Almeida and colleagues propose that emotional resilience plays a protective role in preventing depression in this patient population. Confirmation of these hypotheses would support the need for psycho-oncological interventions aimed at enhancing resilience and reducing emotional vulnerability. This underscores the importance of a multidisciplinary approach that integrates psycho-oncology with biomedical research, offering a more comprehensive perspective on the needs of breast cancer patients.

These considerations support the early integration of psycho-oncological interventions as part of multimodal breast cancer therapy. The primary goal is to mitigate the impact of psychological stress on disease onset and progression. This approach combines relaxation techniques—such as mindfulness, meditation, and guided breathing—with cognitive–behavioral strategies that help reframe maladaptive thoughts and perceptions. Additionally, psychoeducation and emotional support play a key role in addressing stress-related triggers, thereby reducing their potential biological impact.

At the same time, the psycho-oncologist plays a crucial role in identifying and managing emotional distress through early assessment of psychological symptoms in oncology patients undergoing various treatments (by using validated tools, such as the Distress Thermometer developed by the National Comprehensive Cancer Network (NCCN), to identify patients at risk), followed by personalized interventions to address and minimize these symptoms as much as possible.

### 4.1. Early Assessment of Psychological Symptoms in Patients Diagnosed with Breast Cancer

A cancer diagnosis is often perceived as a traumatic event, generating significant emotional impact that can lead to psychological symptoms such as anxiety, depression, post-traumatic stress, or emotional distress. Early assessment of these symptoms is essential for identifying at-risk patients and for implementing timely and effective interventions from the early stages of the disease. Psycho-oncologists play a fundamental role in this process, using validated tools that facilitate the rapid and accurate detection of mental health problems.

Early psychological assessment provides crucial advantages. It allows the timely identification of patients at risk for emotional disturbances and enables prompt, personalized interventions that improve treatment adherence, reduce psychosomatic complications, and enhance overall quality of life by addressing emotional distress.

There are several validated tools available for psychological screening, and one of the most widely used for assessing emotional distress in oncology patients is the Distress Thermometer (DT), developed by the NCCN. This is a simple, self-report instrument that functions as a visual “thermometer” of stress levels. The patient rates their distress on a scale from 0 (no distress) to 10 (extreme distress), based on the past week’s experience. In addition to the numerical scale, the DT includes a “Problem List”, a checklist that helps identify the sources of distress: emotional, physical, social, spiritual, and practical issues (e.g., financial difficulties, family problems, physical pain, anxiety, depression, etc.).

Interpretation of the DT is straightforward: a score of ≥4 is generally considered a warning signal, indicating the need for further evaluation and possible psycho-oncological intervention. The test has multiple advantages: it is quick and easy to administer (takes only a few minutes), cross-culturally valid (used and validated in multiple countries and clinical contexts), and highly sensitive for early detection of distress [24].

Numerous studies support the validity and utility of the DT as a screening tool for identifying psychological distress in oncology patients, with a cutoff score of ≥6 being considered particularly relevant for psychological intervention. For instance, Lisa Graham-Wisener [25] analyzed DT use in a sample of over 200 patients with advanced cancer in palliative care and found that a score of ≥6 was optimal for detecting psychological distress in this population, highlighting its adequate sensitivity and specificity. The authors also identified a cutoff score of ≥4 as optimal for depression screening.

A more recent study from 2023 by Hammoda Abu-Odah, conducted on over 300 patients, confirmed that a DT score of ≥6 is effective in identifying distress in patients with advanced-stage cancer, supporting the use of this threshold in clinical screening [26].

There are also other complementary tools that psycho-oncologists can use: the Patient Health Questionnaire-9 (PHQ-9), used for depression screening, the Generalized Anxiety Disorder-7 (GAD-7), for assessing generalized anxiety disorders, and especially the Impact of Event Scale—Revised (IES-R), which measures post-traumatic stress symptoms [27].

Thus, we can support the idea of integrating psychological evaluation into oncological practice as an ongoing process: screening should not be limited to the moment of diagnosis but should be repeated throughout treatment and into the survivorship phase. This evaluation should be tailored and personalized based on each patient’s specific risk factors (personal history, social support, cancer stage, etc.), as part of multidisciplinary care. The results of the assessment should be discussed within the oncology team to ensure a holistic approach.

The role of the psycho-oncologist in the evaluation process is therefore essential: implementing psychological screening programs in oncology centers, interpreting results and conducting detailed clinical assessments when needed, planning psychotherapeutic interventions for patients at high risk of emotional disorders, and training medical staff to recognize early signs of psychological distress.

### 4.2. The Next Step: Psycho-Oncological Intervention Through Personalized Techniques, Primarily Based on Cognitive–Behavioral Therapy (CBT), Mindfulness Approaches, and Acceptance-Based Interventions

Cognitive–behavioral therapy (CBT) is one of the most widely used and effective forms of psychotherapy for addressing emotional problems in oncology patients. CBT helps patients reframe negative thoughts and develop adaptive coping mechanisms, thereby reducing stress, anxiety, and depression, and improving overall quality of life [28].

CBT is based on the principle that thoughts influence emotions and behaviors, and that changing dysfunctional thoughts can lead to better emotional and behavioral adaptation. In the context of cancer, patients may struggle with persistent negative thoughts such as “I won’t be able to handle the treatment”, “This disease has completely ruined my life”, “I’m a burden to my family”, or “I’m no longer the woman I used to be”. CBT helps patients identify, evaluate, and replace these thoughts with more realistic and constructive ones, thus improving psychological well-being and coping behaviors.

The main CBT techniques used in psycho-oncology focus on cognitive restructuring, which involves identifying automatic negative thoughts (encouraging the patient to recognize thoughts that trigger anxiety or depression), analyzing and challenging these thoughts (assessing whether they are realistic or cognitive distortions), and formulating more balanced alternative thoughts (e.g., instead of “I’m powerless against this disease”, the patient can be guided to say, “I’m doing everything I can to take care of myself and follow the treatment plan”).

CBT also encourages adaptive coping strategies that help reduce physical symptoms of anxiety. These include maintaining enjoyable activities, cultivating present-moment awareness to counteract intrusive worries about the future, and replacing self-criticism with techniques that promote self-compassion and emotional resilience.

A recent study conducted by Lina Xiang [29] investigated the impact of CBT on resilience in adult cancer patients. By analyzing 12 randomized controlled trials, the authors found that CBT had a significant effect on increasing resilience in oncology patients, both immediately after the intervention and at follow-up. Moreover, face-to-face CBT interventions demonstrated a greater impact on resilience compared to online approaches. The study highlights the importance of integrating CBT into the care plan of cancer patients, emphasizing its role in enhancing adaptive capacity and emotional resilience. These findings support the effectiveness of CBT in developing adaptive coping mechanisms among oncology patients, aiding in the reframing of negative thoughts and improving quality of life.

Additionally, a study conducted in 2022 by Zhang L. [30] evaluated the effectiveness of CBT in reducing symptoms of anxiety and depression among breast cancer patients. By analyzing data from 15 randomized controlled trials involving 1979 cancer survivors, the researchers found that CBT significantly reduced depression and anxiety scores immediately post-intervention and up to six months of follow-up. These findings support the recommendation of CBT use among cancer survivors, highlighting its benefits in improving psychological health and quality of life.

Another successful intervention in supporting cancer patients is guided meditation, a deep relaxation technique that is extremely beneficial, offering an effective means of stress reduction, alleviation of psychological symptoms, and support in managing emotions associated with the disease [31,32]. Using a guide (a therapist, a psycho-oncologist, or even an audio recording), guided meditation helps patients focus on their breathing, positive imagery, and reconnecting with their own body. The benefits of guided meditation for cancer patients are numerous.

*Reducing stress and anxiety*: A cancer diagnosis often brings a high level of anxiety and emotional stress. Guided meditation helps calm the nervous system by activating the relaxation response, reducing cortisol levels (the stress hormone), and promoting a sense of calm. Studies have shown that guided meditation can significantly improve emotional well-being and reduce the intensity of negative emotions such as fear or worry.

*Pain relief*: Pain—whether caused by the illness or related treatments—is a common issue for many patients. By focusing on positive mental imagery and redirecting attention away from pain, guided meditation can help decrease pain perception and increase tolerance to discomfort.

*Improved sleep quality*: Insomnia and sleep disturbances are frequently reported by cancer patients. Guided meditation helps relax the mind and body, contributing to deeper and more restful sleep. This practice also helps reduce intrusive thoughts that might otherwise keep patients awake at night.

*Fostering a sense of control and hope*: Guided meditation encourages patients to focus on the present moment and reconnect with their inner resources. By visualizing positive images or scenarios in which they feel healthy and strong, patients can develop a more optimistic attitude toward treatment and life.

*Supporting emotional and physical recovery*: Guided meditation facilitates the release of accumulated emotions and promotes a state of mental balance. It can contribute to a reduction in depressive symptoms and help improve overall well-being.

The practice of guided meditation sessions can be implemented at various stages of medical treatment—before therapy, to reduce anxiety related to invasive procedures or chemotherapy; after therapy sessions, to facilitate physical relaxation and recovery; or as part of a daily ritual, providing a moment of calm and reconnection with oneself.

Clinical studies suggest that guided meditation has a significant impact on reducing depressive and anxiety symptoms, increasing energy levels, and, notably, improving immune function by lowering chronic inflammation. Guided meditation is an accessible, non-invasive, and highly effective technique for supporting breast cancer patients, helping to reduce stress, alleviate pain, and promote general well-being. It can thus become a valuable tool in psycho-oncological care, significantly improving the quality of life of cancer patients. Charalambous A. highlighted the potential benefits of guided imagery and related techniques in supporting breast cancer patients after the first cycle of chemotherapy [33]. The research evaluated the effects of guided imagery on quality of life and psychological symptoms in women with breast cancer. The group practicing guided imagery showed significant improvements in quality of life and reduced anxiety and depressive symptoms.

An innovative psychological approach is narrative therapy, which enables breast cancer patients to reinterpret distressing life events and reconstruct their personal narratives in a way that fosters a renewed sense of agency, coherence, and control over their experiences [34,35]. This form of therapy is based on the premise that individuals create their reality through the stories they tell about their lives, and that reframing stressful experiences can profoundly impact emotional and mental well-being. For women diagnosed with breast cancer, the experience of the diagnosis and subsequent treatments often marks a pivotal, emotionally charged chapter in their personal life stories, influencing their identity, relationships, and outlook on the future. Narrative therapy encourages patients to identify and articulate the emotions, fears, and thoughts associated with these experiences. Through a process known as externalization, patients are guided to separate themselves from the problem (e.g., saying “cancer is a challenge in my life” instead of “my life is defined by cancer”), allowing them to adopt a new, less overwhelming perspective.

This therapeutic approach also focuses on reconstructing personal meaning by identifying internal and external resources that can support healing. For instance, women are guided to reflect on past experiences where they successfully overcame adversity, drawing on those moments as sources of resilience and inner strength to navigate the current challenges of illness. This reinterpretation process enables them to rediscover a sense of meaning and purpose, even amidst a major health crisis such as cancer. Moreover, narrative therapy facilitates the re-authoring of life stories by shifting the dominant narrative from one of victimhood (“Cancer is part of my journey, not my identity”) to one of empowerment (“I am a survivor who takes charge of her life”). This reconstruction of identity has the potential to significantly reduce emotional distress and foster psychological well-being.

Through narrative therapy, an attempt is made to integrate the therapeutic process into patients’ daily lives, by exploring their core values and desires in addition to reinterpreting illness-related experiences. Patients can learn to focus on the positive aspects of their lives and set goals that reflect what truly matters to them. This process can lead to better treatment adherence and an improved quality of life. Studies on narrative therapy show that it can significantly reduce anxiety and depression, helping patients feel more engaged in their own healing journey. Furthermore, this approach provides a sense of control, contributing to greater adherence to treatments and improved relationships with loved ones.

A concrete example might involve a patient who perceives a breast cancer diagnosis as a profoundly impactful diagnosis. Through narrative therapy, she is guided to identify moments of personal courage and integrate them into her story, transforming the cancer experience into a life lesson that strengthened her spirit. Narrative therapy not only supports patients in coping with emotional stress but also offers them the opportunity to reconnect with their inner strength, turning the cancer story into one of growth and resilience. This approach clearly illustrates how personalized psychological interventions can significantly transform patients’ lives.

The study published by Craig D. Blinderman [36] explores the integration of narrative therapy into palliative care, providing clinicians with an additional framework to uncover patients’ core values and guide them in rewriting their stories and addressing challenges. Key aspects discussed in the article include externalization of problems (helping patients distance themselves from their difficulties, allowing them to view these issues as separate entities and reduce self-criticism), identification of unique outcomes (focusing on moments when patients have successfully overcome adversity, thereby highlighting their internal strengths and resources), and re-authoring the personal story (by exploring their values and identity, patients can construct narratives that better reflect who they are and what they value, facilitating a more positive adaptation to illness). Blinderman emphasizes that narrative therapy offers a collaborative, non-pathologizing approach that can enhance well-being and reduce suffering in palliative care patients. Integrating narrative therapy into palliative care practice may support patients in adapting to the reality of illness, offering a safe space for reflection and reconstruction of their personal stories, and ultimately contributing to a more humanized and patient-centered end-of-life experience.

Psychological interventions carried out within support groups also bring real benefits. Participation in support groups led by psycho-oncologists plays a crucial role in assisting breast cancer patients, providing them with a safe space to explore and share their lived experiences [37,38]. These groups significantly reduce social isolation—a common issue among individuals facing an oncological diagnosis—by fostering strong emotional connections with other women experiencing similar challenges. One major benefit of support groups is the creation of a sense of belonging. Through interaction with fellow patients, participants realize they are not alone in their fight against the disease. This awareness offers emotional validation, helping to reduce anxiety, fear of recurrence, and feelings of helplessness. At the same time, psycho-oncologists facilitate these interactions, promoting constructive dialogue and guiding the groups through validated stress reduction techniques, such as progressive relaxation or mindfulness.

Moreover, support groups are a valuable source of practical information. Within these settings, patients can learn about treatment options and side effects, as well as ways to manage them. Psycho-oncologists may introduce relevant topics such as coping techniques, nutrition, or the importance of physical activity, further supporting the adjustment process.

These groups also offer a platform for building psychological resilience. Patients learn to overcome obstacles through the examples shared by fellow group members, which increases their confidence in handling adversity. Studies show that women who participate in support groups report a better quality of life, lower levels of depression, and greater adherence to treatment.

In conclusion, support groups led by psycho-oncologists not only offer emotional refuge but also contribute to enhancing patients’ sense of control over their lives, significantly improving their overall well-being.

An increasing body of literature, including the article by D’Andre and collaborators [39], highlights the complex relationship between chronic stress and cancer. Stress is identified not only as a psychological factor but also as a biological mechanism that contributes to the incidence and progression of breast cancer. The importance of psychological interventions—such as CBT and mindfulness-based stress reduction techniques—is essential in reducing the impact of stress on breast cancer patients. Recent studies show that these techniques not only improve patients’ psychological well-being but also reduce physiological levels of stress, with benefits for quality of life and potentially the prognosis of the disease.

In addition to individual strategies, social support plays a central role. Supportive relationships, whether from family members or support groups, are associated with more positive emotional responses and better adaptation to diagnosis and treatment. Psycho-oncologist-led interventions can facilitate the development of these support networks and improve communication between the patient and the medical team.

The integration of these forms of therapy into oncological care can significantly transform patients’ perception of the illness, offering them a renewed sense of control and hope in the face of adversity.

Building upon the importance of early distress detection, the next section examines how psycho-oncological support contributes to treatment adherence.

## 5. Argument 2: Improving Treatment Adherence

Treatment adherence is a crucial factor in the therapeutic success of oncology patients, significantly influencing both prognosis and quality of life. However, many patients face emotional, cognitive, and social difficulties that can compromise compliance with oncological treatments. Moreover, studies show that anxiety, depression, and negative illness perceptions may lead to treatment discontinuation or reduced adherence. In this context, the role of the psycho-oncologist becomes essential, as psychological interventions can facilitate the acceptance of and commitment to the treatment plan [40].

Oncology patients may encounter numerous barriers that affect their ability to follow the recommended therapies, including a range of emotional, cognitive, and social factors. Among the most impactful emotional factors are anxiety, depression, the quality of the relationship with the attending physician, fear of treatment side effects, and fear of recurrence—all of which may lead patients to avoid or discontinue treatment. Cognitive factors are also frequently involved in decreased adherence and include difficulties in understanding the benefits of therapy or misconceptions about prognosis, poor communication with the medical team, and hypochondriac tendencies often fueled by self-directed online research, which can undermine motivation to continue treatment.

At the same time, various social factors can negatively influence therapeutic adherence. The most cited include lack of family support or disease-related stigma, which may contribute to patient isolation and reduced engagement in the treatment process. Lastly, side effects of the treatment—such as fatigue, nausea, pain, or other symptoms—can also lead to treatment discontinuation.

The psycho-oncologist can play a key role in supporting treatment adherence, having at their disposal several avenues of action [41]. These include, first and foremost, patient education, helping patients better understand the purpose and benefits of oncological treatments, while also reducing fears related to side effects. Psychological barriers are addressed through targeted interventions aimed at reducing fear of recurrence—a frequent obstacle to adherence to maintenance treatments—and managing feelings of helplessness and loss of control; techniques such as narrative therapy support patients in regaining a sense of agency and psychological balance. At the same time, psycho-oncologists can provide ongoing emotional support, collaborating with the multidisciplinary team to monitor the patient’s psychological state and offer personalized support throughout the treatment process.

A compelling example regarding the effectiveness of psycho-oncological interventions in supporting adherence to long-term treatment is provided by the systematic review and meta-analysis conducted by Bright et al. [42]. The analysis included 25 studies, with a total sample of over 367,000 breast cancer survivors undergoing adjuvant endocrine therapy. The results showed a statistically significant effect of interventions on improving treatment adherence compared to control conditions (odds ratio = 1.41; 95% CI: 1.18–1.68; *p* = 0.0001). The evaluated interventions included a variety of components, such as psychological counseling, reminders, educational support, and reduction of cost-related barriers. The authors emphasize that psycho-oncological interventions significantly contribute to maintaining patient motivation, reducing the risk of therapeutic abandonment, and fostering a functional patient–medical team relationship. In this regard, the integration of the psycho-oncologist into the multidisciplinary team appears to be a necessary condition for optimizing adherence to adjuvant hormonal therapy, with a direct impact on long-term clinical outcomes and quality of life.

### 5.1. Medical Education—A Key Element in Improving Treatment Adherence

Medical education, or more specifically psycho-oncological education in this context, is a fundamental component of oncological care, having a direct impact on treatment adherence, anxiety reduction, and quality of life improvement. Psycho-oncologists play a crucial role in this process by helping patients better understand the purpose and benefits of cancer treatments, as well as by addressing fears related to side effects. An informed patient is better equipped to make conscious decisions and to cope with the challenges of therapy [43].

The importance of education in oncology stems from the fact that, for many patients, a breast cancer diagnosis comes with a wave of uncertainty and fear. It is essential to acknowledge the overwhelming emotional turmoil that patients may experience throughout the various stages of treatment. A lack of information or a superficial understanding of treatment options can lead to increased anxiety, resistance to therapy, and even treatment abandonment. Psycho-oncologists intervene to clarify key aspects, turning patients into active partners in their own care journey.

Another essential aspect of patient education lies in reducing treatment-related fears, particularly the fear of side effects, which is one of the most significant barriers to treatment adherence. The most common concerns among breast cancer patients include hair loss, mastectomy and associated body image changes due to chemotherapy, chronic fatigue, the impact of treatment on daily life, pain and other unpleasant symptoms, and potential long-term effects on fertility and overall health [44]. Psycho-oncologists play a key role in helping patients better understand their treatments and normalize their fears by providing clear explanations of what to expect, how to manage side effects, and how to maintain well-being throughout the course of therapy.

Psycho-oncological education relies on several key techniques, including clear and personalized communication about treatments—using accessible language to explain chemotherapy, radiotherapy, and hormonal therapies according to each patient’s level of understanding and needs. It also involves debunking common myths (e.g., ‘Chemotherapy completely destroys the body’ or ‘Radiotherapy makes the body radioactive’) and directing patients toward credible, evidence-based sources of medical information.

Developing coping strategies for side effects is extremely important, including practical advice for managing fatigue, nausea, and other adverse reactions, as well as encouraging patients to maintain a balanced routine of sleep, nutrition, and physical activity. In addition, patients often require support in making informed decisions, with clear explanations of treatment alternatives and their respective impacts. Involving patients in their own decision-making process serves to enhance their sense of control.

The impact of psycho-oncological education on patients is reflected in higher treatment adherence, with patients being less likely to discontinue therapy. They also experience lower levels of anxiety and depression, as understanding their treatment helps to alleviate fears. Ultimately, their quality of life improves, as they acquire effective strategies to cope with challenges [45].

The active communication between the psycho-oncologist and the patient becomes a cornerstone of oncological care, playing a vital role. By providing clear, accurate, and personalized information, the psycho-oncologist helps reduce fear, improve treatment adherence, and enhance quality of life. An informed patient is a stronger, more confident individual, better prepared to face the challenges of illness.

### 5.2. Addressing Psychological Barriers

Addressing the psychological barriers that can affect both the emotional state of patients and adherence to maintenance treatments represents another major challenge for the psycho-oncologist. Most frequently, patients diagnosed with breast cancer face fear of recurrence, feelings of helplessness, and a lack of control over their lives and illness [46,47]. Psycho-oncologists play a crucial role in identifying and managing these difficulties by using scientifically validated interventions to improve patients’ adjustment to life after treatment.

*Regarding the fear of recurrence*, one of the most reported concerns among breast cancer survivors, it significantly impacts their quality of life. This anxiety can lead patients to become hyper-vigilant about minor symptoms, avoid medical check-ups due to anticipatory stress, or, conversely, become excessively preoccupied with screening tests.

Psychological interventions for managing fear of recurrence involve multiple strategies to address this issue. CBT helps patients identify and restructure irrational thoughts related to the likelihood of recurrence, thereby reducing the associated anxiety. A relevant example is provided by the meta-analysis conducted by Fangxin Wei [48]. The study assessed the effectiveness of CBT in reducing recurrence-related fear among oncology patients, analyzing 32 randomized controlled trials with over 3300 participants. It reported a statistically significant effect of CBT compared to control groups (g = –0.65; 95% CI: –0.86 to –0.44; *p* < 0.001). These results support the notion that cognitive–behavioral interventions can substantially reduce emotional distress linked to recurrence, thus not only improving quality of life but also enhancing patients’ active engagement in the therapeutic process. Integrating the psycho-oncologist into the multidisciplinary team is therefore essential for the early identification of recurrence-related fear and for the implementation of personalized intervention strategies.

Another strong argument can be found in the systematic review published by Paperák P. [49], which analyzes the effectiveness of various psychotherapeutic interventions in reducing fear of cancer recurrence among adult oncology patients. The analysis included 13 rigorous studies and highlighted that CBT, mindfulness-based interventions, and acceptance and commitment therapy are the most effective in reducing anxiety related to the potential recurrence of the disease. Among these, “blended CBT” formats, which combine face-to-face sessions with online components, have shown promising results in increasing both accessibility and therapeutic efficacy. The authors emphasize the necessity of tailoring interventions to the patient’s emotional profile and preferences, underscoring the importance of integrating these methods into standard psycho-oncology practice to reduce the impact of fear of cancer recurrence on quality of life and treatment adherence.

Through mindfulness therapy and various progressive relaxation or controlled breathing techniques, patients are taught to stay grounded in the present moment and to manage catastrophic thoughts related to the future [50,51]. Gradual exposure to anxiety-triggering factors is also encouraged; for instance, some patients may avoid hospitals or conversations about cancer, and psycho-oncologists can help them face their fears progressively, thus reducing intense emotional reactions. In this regard, Rachel Telles [52] investigated the effectiveness of mindfulness-based psychosocial interventions (MBIs) on the psychological well-being of cancer survivors. This meta-analysis of randomized controlled trials assessed the impact of MBIs on positive aspects of psychological well-being among patients with various cancer types, showing that MBIs have a significant positive effect, including improvements in areas such as self-acceptance, personal growth, and interpersonal relationships. These findings underscore the potential of MBIs as valuable interventions for supporting the psychological recovery of cancer survivors, highlighting the importance of holistic approaches in cancer care.

Equally important is the provision of social support through participation in support groups, where sharing experiences with other survivors can help normalize fear of recurrence and provide effective coping strategies. Involving family in the therapeutic process can also increase treatment adherence by offering the patient a stable and motivating support system.

*Psycho-oncological intervention is also necessary for patients who develop a deep sense of helplessness and loss of control* over their lives, following an oncological diagnosis, by helping them manage these emotions [53]. Many patients feel that the illness has taken away their autonomy and that their future is uncertain. These feelings can lead to passivity, avoidance behaviors, or depression, negatively impacting their adjustment to maintenance treatments.

Effective techniques used by psycho-oncologists to restore the sense of control include narrative therapy, self-efficacy-focused interventions, empowerment-based activities, and acceptance and commitment therapy. Narrative therapy encourages patients to rewrite their personal story, shifting from the narrative of a “victim of illness” to that of a “strong survivor”. This process allows them to redefine their experience and identify the inner resources that have helped them overcome difficult moments, building a more positive perspective on their therapeutic journey.

A relevant study by Chen Geng [54] evaluated the effectiveness of Narrative Exposure Therapy (NET) in treating depressive and anxiety disorders. The meta-analysis included 11 randomized controlled trials involving a total of 754 participants. The authors concluded that NET is effective in reducing symptoms of depression and anxiety, supporting its clinical use in alleviating negative emotional states and promoting general psychological recovery. These findings highlight the potential of NET as a therapeutic intervention for stress-related disorders, offering a valuable alternative in the psychological treatment of cancer patients suffering from distress.

Self-efficacy-focused interventions aim to help patients regain control over manageable aspects of their lives by encouraging the setting of small and realistic goals (e.g., adopting a healthy lifestyle, attending scheduled medical check-ups). Through empowerment-based activities, patients are encouraged to engage in volunteering, express emotions through art therapy, or keep a therapeutic journal—methods proven to support the recovery of personal autonomy and agency.

Acceptance and commitment therapy also plays a key role, helping patients embrace the inherent uncertainty of post-cancer life while reconstructing their identity in a positive, value-driven way.

The impact of these well-structured psychological interventions—rooted in psychotherapeutic, educational, and motivational techniques—can significantly transform the survivorship experience [40,55]. They support women in managing fear of recurrence, regaining a sense of control, developing effective coping strategies, and integrating the cancer experience into their life narrative.

Studies have shown that breast cancer survivors who receive psycho-oncological support report significantly lower levels of anxiety and depression, improved quality of life, higher self-efficacy, better adherence to maintenance treatments, and a more positive outlook on the future. Therefore, psycho-oncological support becomes a cornerstone in the post-treatment adaptation process, contributing to long-term emotional health, increasing the likelihood of therapeutic success, and potentially reducing the risk of recurrence.

Giampaolo Perna and collaborators [16] not only emphasize the importance of a patient-centered approach in personalized medicine but also advocate for the active involvement of patients in treatment-related decisions and the respect for their individual values and preferences. This holistic approach can strengthen the therapeutic alliance and improve treatment adherence, providing further evidence for the crucial role of psycho-oncologists in supporting and educating oncology patients to maintain adherence to prescribed treatments and reduce fears related to their adverse effects.

These findings highlight the effectiveness of psychological interventions, such as cognitive–behavioral therapy, narrative therapy, and mindfulness techniques, in addressing the psychological barriers encountered by breast cancer patients. The implementation of such strategies can significantly enhance quality of life and promote adherence to therapy.

### 5.3. The Importance of Repeated Monitoring of the Patient’s Psychological State

Emotional suffering often remains unaddressed during oncological treatment. Psychological stress can negatively impact the health of patients, affecting treatment response (as anxiety and depression can impair immune system function), treatment adherence (patients experiencing severe emotional distress are more likely to interrupt chemotherapy or refuse maintenance therapies), and quality of life (feelings of helplessness and lack of support can lead to social isolation and decreased self-confidence). Therefore, it becomes essential for psycho-oncologists to intervene through the periodic evaluation of patients’ emotional health, using tools such as anxiety and depression scales or clinical interviews to detect early signs of emotional distress—beginning at the time of diagnosis, throughout treatment phases, and even after the completion of therapy and during follow-up monitoring.

Collaboration between the psycho-oncologist and the multidisciplinary team is crucial, as oncological care requires a comprehensive and patient-centered approach. Psycho-oncologists contribute significantly by coordinating with oncologists and nurses to ensure the integration of psychological support into the treatment plan, delivering personalized interventions for patients struggling with anxiety or depression, and assisting the team in maintaining clear and compassionate communication. Equally important, they provide empathetic guidance to both patients and their families throughout all stages of cancer care, alleviating fears related to treatment side effects and uncertainty about prognosis.

The role of the psycho-oncologist in the multidisciplinary team is crucial in offering cancer patients consistent and personalized emotional support. By monitoring psychological well-being, collaborating with the medical team, and implementing interventions tailored to each patient’s needs, psycho-oncologists contribute to improving both quality of life and therapeutic outcomes [56]. Integrating this type of support into modern oncological practice is essential for a holistic and human-centered approach to the care of breast cancer patients.

## 6. Argument 3: Improving Quality of Life

Quality of life is a key indicator of the success of oncological treatment, especially in the case of breast cancer patients, who face significant physical and emotional changes. Cancer represents one of the most profoundly challenging life experiences, not only affecting physical health but also deeply impacting emotional, social, and psychological well-being. The quality of life of oncology patients is influenced by multiple variables, including treatment side effects, emotional distress, uncertainty about the future, and lifestyle changes.

In this context, the psycho-oncologist plays an important role in improving patients’ quality of life through personalized interventions that offer emotional support, coping strategies, and resources to help them deal with the illness in an adaptive way, by holistically addressing the patients’ needs.

The notion of “quality of life” in oncology represents a multidimensional concept that includes physical status (energy level, pain control, treatment side effects), emotional well-being (anxiety, depression, fear of recurrence, stress level), social functioning (interpersonal relationships, support from family and friends), autonomy, and the ability to carry out daily activities (degree of independence and sense of control over one’s life). The psycho-oncologist intervenes in each of these areas, offering structured support to improve the patient’s adaptation to the reality of the illness.

Anna Lewandowska [57] examines the impact of chemotherapy on the quality of life of oncology patients. The study highlights that cancer has a significantly negative impact on patients’ quality of life, influenced by the disease process, treatment types, and illness duration. Key findings included major difficulties in self-care (81% of patients reported significant self-care challenges) and the presence of anxiety and depression symptoms (63% of patients experienced these issues). These results emphasize the need for psycho-oncological interventions that address the emotional and psychological aspects of patients undergoing chemotherapy, by integrating psychological counseling and supportive therapies into the treatment plan—thus potentially improving treatment adherence and overall quality of life.

### 6.1. The Contribution of the Psycho-Oncologist to Improving Quality of Life Can Be Directed Across Three Important Levels

First, psychological side effects can be managed by reducing the negative impact of stress and anxiety associated with diagnosis and treatment, and by supporting patients in adapting to body image and identity changes (e.g., hair loss, mastectomy). Second, a balanced lifestyle can be promoted through interventions focused on developing coping strategies such as relaxation techniques, breathing exercises, and encouraging the integration of physical activity and healthy nutrition into daily routines. Third, social support can be strengthened by facilitating communication between patients and their families and by creating support networks through therapeutic groups [58,59].

*Regarding the management of psychological side effects*, a primary focus is the reduction in the negative impact of stress and anxiety associated with diagnosis and treatment. A breast cancer diagnosis is often perceived as a traumatic event, generating high levels of anxiety, depression, and stress. These emotional reactions are amplified by uncertainty regarding prognosis, treatment side effects, and the impact of the illness on daily life.

Psycho-oncologists intervene through various methods. Cognitive–behavioral therapy helps patients manage catastrophic thinking and replace it with more balanced perspectives. For example, a patient who fears that “she will never be healthy again” can learn to reframe this thought more realistically, such as “I will face challenges, but there are effective treatments that can help me.”

A remarkable example of the importance of psycho-oncology in improving the quality of life for oncology patients is provided by the study conducted by Monica Licu [60]. The paper emphasizes that systematic assessment of quality of life, along with psycho-oncological interventions adapted to cultural and contextual factors, is an essential component of modern oncology practice. The authors highlight the urgent need for a coherent national framework to integrate psychological support as part of standard oncology treatment, especially in countries such as Romania, where cancer mortality rates are above the European average. Among the benefits of psycho-oncology, the study lists reduced depression and anxiety, increased treatment adherence, and an overall improvement in patient well-being. Thus, the integration of the psycho-oncologist into the multidisciplinary team is supported as a necessary step for truly patient-centered care.

Relaxation and mindfulness techniques are also successfully used, as they reduce the physiological responses to stress, such as muscle tension, insomnia, and anxiety-related eating disorders [61,62]. Other methods include gradual exposure therapy, which helps patients confront their fears related to treatment and hospital settings, thereby reducing anticipatory stress associated with chemotherapy or surgical interventions.

Participation in support groups is equally important, providing a safe environment where patients can share their experiences and receive support from other women who have gone through similar challenges.

At the same time, the goal is to increase autonomy and improve illness perception, as many cancer patients feel they have lost control over their lives due to the disease. This is achieved through the establishment of achievable goals, which give patients clear direction and motivation for the future, and the promotion of self-efficacy by identifying internal resources and building confidence in their ability to cope with challenges.

Studies show that these groups significantly reduce anxiety and improve overall psychological well-being, as evidenced by Sunre Park [63]. The authors demonstrated the effectiveness of mindfulness-based cognitive therapy in supporting breast cancer patients. After eight weeks of intervention, participants showed a significant reduction in psychological distress, fatigue, and fear of recurrence, along with improvements in quality of life and spiritual well-being. These positive effects were maintained even in the post-intervention assessment, supporting the integration of mindfulness-based cognitive therapy as an effective tool in the holistic psycho-oncological approach for cancer patients.

Equally important is the psycho-oncologist’s role in supporting patients as they adapt to the bodily and identity-related changes resulting from breast cancer treatment. Such treatment often leads to significant physical transformations that can impact self-image and personal identity. Hair loss, mastectomy, and postoperative scars may profoundly affect self-confidence, interpersonal relationships, and intimacy.

Psycho-oncologists guide patients through this adjustment process using tailored interventions. Particularly useful tools include narrative therapy and identity reconstruction, where patients are encouraged to reframe their personal stories—shifting from a perception of being a “victim of the disease” to that of a “resilient survivor”. Expressive writing and therapeutic journaling are often used to help patients process emotions and redefine their identity.

Cognitive restructuring techniques aimed at accepting bodily changes support patients in reshaping their body image, helping them develop a more positive perception of their post-treatment body. They are encouraged to focus on the functional aspects of the body rather than on the aesthetic changes, although reconstructive surgery techniques come to compensate for these problems [64].

These body image concerns are also highlighted by Michelle Cororve Fingeret [65]; the author emphasizes that oncology patients—particularly women who undergo surgeries or treatments that alter physical appearance—often experience body image disturbances that can negatively impact self-esteem, social relationships, and overall quality of life. The study recommends the systematic evaluation of body image distress in clinical settings and underscores the effectiveness of cognitive–behavioral therapy in addressing these issues.

Thus, psycho-oncologists play a crucial role in facilitating the acceptance of the new body image and in supporting the emotional adjustment process following cancer treatment.

Another major goal for psycho-oncologists in improving the quality of life of oncology patients is the promotion of a balanced lifestyle. A healthy lifestyle is important in the recovery process of oncology patients, helping to reduce stress levels, improve overall health, and increase psychological resilience. Psycho-oncologists contribute to the adoption of a balanced lifestyle through interventions that include the development of coping strategies and the integration of healthy habits—essential measures to help patients manage intense emotions and the challenges associated with illness and treatment.

A range of techniques are employed to enhance patients’ adaptive capacity. These include relaxation methods—such as progressive muscle relaxation, autogenic training, and guided imagery—which help alleviate stress and improve sleep quality. Additionally, breathing exercises like diaphragmatic and rhythmic breathing are effective in reducing anxiety and promoting calm during stressful situations, such as awaiting medical results. Mindfulness practices, meditation, and acceptance and commitment therapy (ACT) further support emotional acceptance and encourage patients to pursue meaningful, value-based goals.

Studies show that mindfulness practices help reduce cortisol levels (the stress hormone) and improve emotional regulation in oncology patients. All these interventions contribute to reducing anxiety, enhancing emotional control, and increasing patients’ psychological comfort [66,67].

Equally important is supporting the integration of physical activity and healthy eating habits, as adopting a healthy lifestyle is essential in the post-oncological recovery process. Psycho-oncologists can play an active role in motivating patients to adopt beneficial habits by collaborating with nutritionists and physiotherapists to develop a personalized plan. The importance of moderate physical activity—such as yoga, Pilates, or daily walks—lies in its ability to improve patients’ mobility, energy, and emotional balance. Regular physical activity has been associated with a reduced risk of cancer recurrence and better overall quality of life.

At the same time, nutrition is closely linked to emotional well-being. A balanced diet helps patients maintain optimal energy levels and supports immune system function. Psycho-oncologists can contribute to the development of healthy eating habits by helping patients reduce emotional eating and by promoting balanced nutritional choices. Through the promotion of physical activity and balanced nutrition, patients may experience faster recovery, improved mood, and reduced chronic fatigue.

All these ideas are explored in the qualitative study conducted by Lynda G. Balneaves [68], which examines the experiences of women with breast cancer and their oncologists regarding lifestyle interventions recommended during adjuvant treatment. The results highlighted that lifestyle recommendations made by the treating oncologist had a significant positive impact on patients’ motivation to adopt beneficial changes such as regular exercise and a balanced diet. Participants reported physical, psychological, and social improvements, while oncologists observed a strengthened doctor–patient relationship. The study emphasizes the essential role of the medical team in supporting lifestyle changes integrated into cancer care as part of a patient-centered approach.

*Quality of life is also measured through the lens of social relationships*, with psycho-oncologists playing an important role in strengthening social support for oncology patients by improving interpersonal connections and facilitating social reintegration. It is well known that cancer can lead to social isolation, both due to the stigma associated with the disease and the patient’s difficulty in reintegrating into an active social life. Social relationships and support from family and friends are essential factors in improving the quality of life of oncological patients [69,70]. Psycho-oncologists contribute to strengthening these support networks by facilitating effective communication and organizing support groups.

They can also mediate communication between patients and their families, since a cancer diagnosis affects not only the patient but the entire family system. Often, patients may feel that their loved ones do not fully understand what they are going through or that they do not know how to express their emotions and needs. Psycho-oncologists assist in fostering open communication between patients and their families, reducing stress and relationship tensions [71,72,73].

Key interventions include many directions, including supporting life partners in coping with relationship changes, especially regarding physical changes following surgery. Emotional support is offered to help couples readjust to intimacy and their shared life, as many patients experience fear of rejection or face relationship difficulties following oncological treatments. In response to these challenges, surgical techniques have also been developed in the context of genital cancers, with a focus on preserving fertility whenever possible [74]. Psycho-oncologists facilitate open dialogue between partners and offer strategies for rebuilding self-confidence, guiding family members on how to offer effective support. Although loved ones often have good intentions, their actions may unintentionally add stress. The psycho-oncologist helps them better understand how to be more empathetic and genuinely supportive. Open communication reduces the patient’s emotional isolation, improves relationships, and enables more effective support from the family. Likewise, psycho-oncologists promote the involvement of family and life partners in the therapeutic process to enhance emotional support and communication. They also help develop communication strategies that empower patients to clearly express their needs and boundaries to those around them.

Equally important is the intervention aimed at creating support networks through therapeutic groups, as support groups play a crucial role in helping patients normalize their emotions and learn effective coping strategies [37,75]. Psycho-oncologists organize and facilitate such groups, which offer significant benefits: reducing the feeling of isolation (patients learn they are not alone in this experience and that there are others who share similar emotions and challenges), sharing coping strategies (discussions with other patients can provide practical solutions and new perspectives on managing the disease), and increasing hope and resilience (the inspirational stories of survivors can have a profound impact on women undergoing treatment).

Following the completion of oncological treatment, many patients experience fear of recurrence, uncertainty about the future, and challenges in resuming their daily routines—making support during the post-treatment adjustment period essential. Psycho-oncologists play a key role by offering strategies to manage fear of recurrence, such as gradual exposure and cognitive restructuring, as well as counseling for reintegration into social and professional life by identifying accessible resources. Additionally, they guide patients through post-treatment care programs aimed at promoting long-term well-being.

### 6.2. The Psycho-Oncologist’s Approach to the Concept of “Rebirth Through Cancer”

At the same time, the psycho-oncologist’s approach to the concept of “rebirth through cancer” aligns perfectly with their role in transforming a traumatic experience into an opportunity for personal growth [76,77]. Under the guidance of the psycho-oncologist, patients can find new meaning in life, redefine their priorities, and build a more positive outlook on the future. Thus, although the diagnosis of cancer is often perceived as a turning point in patients’ lives—generating feelings of fear, uncertainty, and loss of control—under the psycho-oncologist’s guidance, this experience can become an opportunity for rebirth and personal redefinition.

The concept of “rebirth through cancer” refers to the process by which patients manage to transform the oncological experience into an opportunity for personal and spiritual growth. This rebirth primarily involves rediscovering the meaning of life—many patients develop a renewed appreciation for life, shift their priorities, and focus more on authentic relationships and activities that bring them joy [78]. This process also involves a shift in perspective—as guided by the psycho-oncologist, women can come to see the illness not merely as a battle, but as a journey of self-discovery and personal growth. Furthermore, it entails personal empowerment—rebirth through cancer also means developing a deep sense of self-efficacy, in which the patient is given the necessary tools to regain control over her own life.

Post-traumatic growth (PTG) refers to the positive psychological transformations that breast cancer survivors may experience after confronting the illness. Coping strategies and psychological support play a crucial role in facilitating this process of growth. Studies show that action-oriented coping strategies, such as actively engaging in treatment and seeking social support, are associated with higher levels of PTG among women diagnosed with breast cancer. For instance, one study demonstrated that adopting active coping at the beginning of treatment predicts a significant increase in PTG after 9 months, which subsequently contributes to better physical quality of life at 15 months. A longitudinal study conducted by Amy R. Senger [79] makes a valuable contribution to understanding the relationship between coping mechanisms, post-traumatic stress, and PTG in women with breast cancer. The primary aim of the research was to examine how coping strategies and initial psychological stress influence the development of PTG and its impact on quality of life 15 months post-diagnosis. The study evaluated participants at three key time points: at the start of treatment (T1), at nine months (T2), and at 15 months (T3). The results revealed that active coping strategies adopted early on (e.g., actively engaging in treatment, seeking support, cognitive reframing) were associated with higher PTG levels at nine months. In turn, PTG positively mediated the relationship between coping and health-related quality of life at 15 months, indicating a sustained beneficial effect of these adaptive mechanisms. Another important aspect of the study was the role of cancer-related intrusive thoughts (as indicators of post-traumatic stress), which predicted lower levels of PTG, particularly among participants with low levels of active coping.

These findings suggest that the absence of effective coping strategies may hinder positive transformation processes after illness and negatively affect overall well-being. The authors conclude that promoting active coping and managing post-traumatic stress are essential for facilitating PTG and improving the quality of life in women with breast cancer. The clinical implications of these results emphasize the importance of early psychological interventions focused on developing personal resources, supporting the re-signification of the illness, and strengthening emotional adaptability.

In this regard, psycho-oncologists employ a range of psychotherapeutic techniques and interventions to support this process of transformation. These interventions not only help to alleviate emotional distress but also create opportunities for personal growth and improved quality of life. Narrative therapy helps patients rewrite and restructure their personal story, shifting from a narrative of “cancer victim” to that of a “strong survivor”. A patient who feels overwhelmed by the disease may be encouraged to reflect on past moments when she overcame other challenges, thus reconstructing a positive and resilient self-image.

Mindfulness and acceptance-based techniques are used to anchor patients in the present moment, helping them to focus on the “here and now” and reduce anxiety related to recurrence or future uncertainties. Another effective strategy is helping patients understand and accept illness as part of life—rather than viewing cancer as a disruption of normalcy, they are guided to integrate the experience into their personal journey, finding meaning even in difficult times.

Supporting the exploration of life’s meaning (a concept known as logotherapy) can be fundamental [80]. Discovering a renewed sense of purpose, whether through creative activities or the development of meaningful personal projects, can provide patients with a sense of fulfillment [81]. Engagement in volunteering or mentoring other patients can also offer a powerful sense of usefulness and meaningful contribution, reinforcing personal identity and emotional strength.

An eloquent example in this regard is the study conducted by Maria Luisa Martino and Maria Francesca Freda [82], which highlights the role of narrative processes in facilitating post-traumatic growth among cancer survivors. The analysis of narratives showed that patients who reflect on their oncological experience from a transformative perspective develop more solid coping mechanisms and a renewed sense of life meaning. The authors distinguish between reflection-centered transformation—associated with a profound restructuring of self-perception and worldview—and action-centered transformation, oriented toward reorganizing daily life. The results emphasize the importance of psycho-oncological interventions based on narrative therapy, capable of supporting patients in the process of re-signifying illness and reconstructing their identity. The objectives of the study were to identify narrative markers that indicate PTG processes in cancer survivors and to analyze the transformative functions of these markers in patient narratives. The study used a mixed methodology, combining quantitative and qualitative analyses, based on the narratives of 12 cancer survivors, divided into two groups: one with medium/high levels of PTG and another with medium/low levels. In narratives from the high/medium PTG group, a reflection-centered transformation was observed, characterized by changes or expansion of internal interpretive frameworks of their relationship with the world, with a focus on the present and future self. In contrast, narratives from the medium/low PTG group showed an action-centered transformation, marked by operational changes in how to live in the present moment. The study underscores the importance of narrative markers in understanding post-traumatic growth processes in cancer survivors. The identified transformative functions can guide clinical interventions aimed at supporting patients in adapting and growing personally after the oncological experience.

The benefits of “rebirth through cancer” in terms of quality of life for patients who manage to transform the oncological experience into personal growth include lower levels of anxiety and depression, due to a shift in perspective on the disease; an improved quality of life, through conscious living and the appreciation of positive moments; deeper social relationships, based on authenticity and mutual support; and enhanced psychological well-being, marked by increased self-efficacy and emotional resilience. Rebirth through cancer, guided by the psycho-oncologist, represents a powerful path of personal transformation in which patients not only survive the disease but also rediscover joy and meaning in life. Through emotional support, validated therapeutic strategies, and the encouragement of a positive outlook on the future, psycho-oncologists play a key role in improving the quality of life of cancer patients, offering them a new chance at a fulfilling life.

All these interventions contribute to reducing depression, increasing self-confidence, and enhancing the overall quality of life, enabling patients to continue their lives in a more balanced and satisfying way.

### 6.3. A Spiritual Resource in Support of Breast Cancer Patients, Mediated by the Psycho-Oncologist, Can Also Be the Connection with the Divine

Offering comfort, meaning, and hope during difficult moments. In the context of psycho-oncology, addressing spirituality and faith can significantly contribute to improving quality of life and facilitating emotional healing and personal rebirth. A valuable contribution to understanding the role of the spiritual dimension in supporting cancer patients is brought by the study conducted by Dana Sonia Nagy [83], which investigates the influence of spirituality and religion on quality of life and coping mechanisms in patients diagnosed with cancer. The results show that a higher level of spirituality is correlated with a significant reduction in symptoms of anxiety and depression, along with enhanced coping strategies and psychological resilience. Religious practices and faith provide patients with a framework of meaning, emotional support, and a source of hope, thus contributing to a more positive perception of life and better adaptation to illness. The authors emphasize the need to integrate spiritual support into the oncological therapeutic plan, recommending that healthcare professionals be receptive to the spiritual needs of patients and, where appropriate, collaborate with spiritual counselors or representatives of religious communities.

The cancer experience can trigger an existential crisis, in which questions related to the meaning of life, suffering, and death become central. In such moments, connecting to the Divine can offer profound support, helping patients find inner peace and hope. Psycho-oncologists can facilitate this process while always respecting the individual beliefs and values of their patients [84,85].

The role of spirituality in the healing process can be significant. Spirituality is not limited to religion; rather, it involves a personal search for meaning in life, inner peace, and a connection to something greater than oneself. For oncological patients, connecting to the Divine can offer emotional refuge in the face of uncertainty and fear. Practices such as prayer, spiritual meditation, and religious rituals contribute to reducing stress and anxiety by providing an anchor in the present moment. Additionally, belief in a higher power can enhance feelings of trust and hope, helping patients to view illness as part of a larger plan—one that may be difficult to understand, but which can hold deep personal meaning [86,87].

Psycho-oncologists can support patients in exploring and strengthening their spiritual resources through techniques inspired by existential therapy and logotherapy (developed by Viktor Frankl), focusing on the search for meaning in life and transforming difficult experiences into opportunities for personal growth. A patient who asks herself “Why is this happening to me?” may be guided to reframe the question as “What can I learn from this experience?” or “How can I use this situation to help others?”

Additionally, integrating spiritual practices into daily routines, especially prayer and guided meditation, can become moments for reflection and spiritual connection, according to the patient’s own spiritual tradition. Another valuable tool is guided imagery, using positive mental images, such as visualizing oneself surrounded by healing light or connected to a higher energy, to bring inner peace and calm.

Depending on the patient’s wishes, psycho-oncologists can also collaborate with priests, pastors, rabbis, imams, or other spiritual leaders to provide integrated spiritual support [88].

The benefits of connecting with the Divine on the quality of life of some patients are worth considering, as international studies show that spirituality can have a significant positive impact on cancer patients. Faith in a higher power can offer a sense of safety and protection, reducing fear of death and uncertainty about the future. Spirituality contributes to the reduction of stress and anxiety, as well as to the improvement of emotional well-being—patients who engage in prayer or spiritual meditation often report lower levels of depression and greater emotional resilience.

Connecting with the Divine can also help patients develop a deeper sense of life purpose, which positively influences their social relationships, treatment adherence, and outlook on the future.

To synthesize the key psycho-oncological interventions and their demonstrated outcomes in breast cancer care, we compiled the following summary table (Table 1). It outlines the most frequently utilized therapeutic approaches, associated psychological and clinical benefits, and corresponding evidence from recent literature.

## 7. Limitations of the Review

While the evidence compiled in this review highlights the effectiveness of psycho-oncological interventions in breast cancer care, certain limitations must be acknowledged. Many of the included studies varied in methodology, sample size, and follow-up duration, which limits the comparability of outcomes. Furthermore, not all studies employed rigorous controls or assessed risk of bias using standardized tools (e.g., GRADE or Cochrane frameworks), which may affect the reliability of reported results. Publication bias cannot be excluded, as studies with positive findings are more likely to be published. Additionally, heterogeneity in psycho-oncological interventions and patient populations may influence outcome generalizability. Despite these limitations, the overall trends consistently suggest beneficial effects across psychological and treatment-related domains.

One of the main limitations encountered in the reviewed literature is the scarcity of quantitative data presented as exact statistical percentages. While many studies document improvements in psychological outcomes such as anxiety, depression, or distress, these effects are often reported using heterogeneous assessment tools and without standardized quantification. Most studies tend to isolate specific psychological variables, making it difficult to aggregate or compare results across different interventions. As such, while the general efficacy of psycho-oncological interventions is supported qualitatively and through selected measures, the lack of comprehensive statistical reporting limits broader generalizations regarding the magnitude of these benefits.

## 8. Future Research Recommendations

To strengthen the evidence base and allow for more robust comparisons, future studies should aim to standardize psychological assessment tools and report effect sizes or percentage-based improvements where possible. The use of validated instruments across larger, more diverse cohorts would enable meta-analyses and clearer estimations of the impact of psycho-oncological interventions. Additionally, longitudinal designs assessing psychological outcomes over extended periods post-treatment could provide valuable insight into the sustained benefits of such support.

## 9. Conclusions, Interpretation of Benefits, and Recommendations

### 9.1. Interpretation of Benefits

The literature consistently demonstrates that psycho-oncological interventions—ranging from cognitive–behavioral therapy, mindfulness-based stress reduction, and narrative therapy, to guided imagery—offer measurable benefits across several domains:

- Psychological well-being: Reduced symptoms of anxiety, depression, and fear of recurrence.
- Treatment outcomes: Increased adherence to hormonal and chemotherapeutic regimens.
- Quality of life: Enhanced emotional resilience, body image acceptance, coping capacity, and post-traumatic growth.
- Long-term survivorship: Improved integration of cancer experience, return to daily activities, and existential adaptation.

Moreover, psycho-oncologists serve as key communicators between the medical team and the patient, improving medical decision-making and empowering patients with a renewed sense of control and meaning.

### 9.2. Recommendations

Given the extensive evidence presented, the integration of psycho-oncological services into standard cancer care is no longer optional but imperative. We recommend the following:

- Systematic psychological screening for all breast cancer patients, starting at diagnosis and continuing throughout treatment and follow-up.
- Routine inclusion of psycho-oncologists in multidisciplinary teams across oncology departments.
- Development of national protocols that mandate psychological support as part of oncological treatment pathways, especially in countries with limited psycho-oncology infrastructure.
- Further research into culturally adapted, cost-effective psycho-oncological interventions that can be implemented at scale, particularly in low- and middle-income countries.
- Educational programs for oncology staff, to improve emotional literacy and facilitate timely referral of patients to psycho-oncology services.
- Short-term: Integrate psycho-oncological screening into diagnostic pathways and train clinicians to recognize emotional distress.
- Medium-term: Establish multidisciplinary care teams including psycho-oncologists; implement evidence-based psychological interventions.
- Long-term: Develop national psycho-oncology strategies, secure sustainable funding, and promote research on culturally adapted interventions.

### 9.3. Conclusions

This rapid narrative review highlights the essential role of psycho-oncologists in the comprehensive care of breast cancer patients. The psychological burden accompanying the diagnosis and treatment of breast cancer significantly influences patients’ mental health, treatment adherence, and quality of life. By identifying and addressing emotional distress early, improving compliance with adjuvant therapies, and supporting personalized psychological resilience strategies, psycho-oncologists contribute decisively to a more holistic and effective oncological care pathway.

### 9.4. Final Reflection

Cancer is not solely a medical battle—it is a profound human journey. Behind every diagnosis stands a woman confronting uncertainty, fear, and transformation. Psycho-oncologists do not aim to eliminate the pain, but to walk alongside these patients, helping them reframe their experience: to find resilience in vulnerability, meaning in suffering, and hope amid healing. By uniting scientific insight with compassionate presence, psycho-oncology redefines cancer care—not as a path that ends with survival, but as one that continues through personal renewal. When we acknowledge the psychological dimensions of illness, we move beyond treating the disease to truly caring for the person.

## Figures and Tables

**Table 1:** Summary of psycho-oncological interventions and associated benefits.

Intervention	Reported Benefits/Outcomes	Supporting Evidence
Cognitive–Behavioral Therapy (CBT)	↓ Anxiety and depression; ↑ Treatment adherence	Wei et al., 2024 [48]; Paperák et al., 2022 [49]
Mindfulness-Based Stress Reduction (MBSR)	↓ Stress; ↓ Fear of recurrence; ↑ Emotional resilience	Telles et al., 2024[52] ; Park et al., 2020 [63]
Narrative Therapy	↑ Self-efficacy; Improved identity reconstruction	Martino & Freda, 2016 [82]; Geng et al., 2024 [54]
Acceptance and Commitment Therapy (ACT)	↑ Acceptance; ↓ Distress from uncertainty	Senger et al., 2023 [79] ; Park et al., 2020 [63]
Spiritual Counseling	↑ Sense of meaning; ↓ Existential anxiety	Nagy et al., 2024 [83]
Support Groups	↓ Isolation; ↑ Coping strategies and social connectedness	Senger et al., 2023[79]; Park et al., 2020 [63]
Psychoeducation	↑ Treatment understanding; ↓ Fear of side effects	Bright et al., 2023 [42]
Logotherapy	↑ Purpose and life satisfaction	Viktor Frankl

## Data Availability

No new data were created or analyzed in this study.

## Abbreviations

The following abbreviations are used in this manuscript:

HPA	hypothalamic–pituitary–adrenal
VEGF	vascular endothelial growth factor
CBT	cognitive–behavioral therapy
PSS	Perceived Stress Scale
HADS	Hospital Anxiety and Depression Scale (HADS)
NCCN	National Comprehensive Cancer Network
DT	Distress Thermometer
MBIs	mindfulness-based psychosocial interventions
NET	Narrative Exposure Therapy
PTG	post-traumatic growth
